# Pupillary responses to bright and dark stimuli in individuals with autism spectrum disorders

**DOI:** 10.1371/journal.pone.0319406

**Published:** 2025-04-01

**Authors:** Tomoe Hayakawa, Shun Nakano, Naoko Inada, Ayako Saneyoshi, Masaki Tsujita, Shinichiro Kumagaya, Naoto Hara

**Affiliations:** 1 Department of Psychology, Teikyo University, Hachioji, Tokyo, Japan,; 2 Research Center for Advanced Science and Technology, the University of Tokyo, Meguro-ku, Tokyo, Japan,; 3 Department of Orthoptics and Visual Sciences, International University of Health and Welfare, Otawara, Tochigi, Japan; Technical University of Munich: Technische Universitat Munchen, GERMANY

## Abstract

Individuals with autism spectrum disorders (ASD) often exhibit difficulties in sensory processing, including visual hypersensitivity such as photophobia. This study investigates the neural mechanisms underlying photophobia in participants with ASD by analyzing pupillary responses. To achieve this, we examined the amplitude and velocity gradient (latency) of these responses. Pupillary responses were recorded using an eye-tracking system in participants with ASD (n =  17) and typically developing (TD) (n =  23). Stimuli alternated between bright (89.03 cd/m^2^) and dark (0.07 cd/m^2^) conditions following a dim state (2.75 cd/m^2^) with intervals of five seconds in Experiment 1 and 30 seconds in Experiment 2. The sensory profile test (AASP-J) showed that hypersensitivity was significantly defined in the ASD group than in the TD group. The pupillary response in the ASD group often featured missing values due to blinking during rapid alternation between bright and dark conditions, resulting in a decrease in the total number of participants. Specifically, only eight of the 17 participants in the ASD group and 20 of the 23 participants in the TD group remained for analysis in Experiment 1, and in Experiment 2, 15 of the 17 participants in the ASD group and 20 of the 23 participants in the TD group remained for analysis. In the dim state, pupillary diameter was large in the ASD and TD group in both experiments, while the pupil diameter decreased in the TD group in Experiment 2. In both experiments, maximum amplitude and its latency showed no significant differences between the two groups. However, the velocity gradient for the early mydriatic process in the dark condition was significantly faster in the ASD group. ASD individuals with hypersensitivity tend to have large pupil diameters under the dim state, as well as rapid dilation in the dark condition. These results may suggest a problem in the sympathetic nervous system, which controls pupil constriction.

## Introduction

Autism spectrum disorder (ASD) is a complex neurodevelopmental disability characterized by deficits in social communication and social interaction across multiple contexts, as well as repetitive patterns of behavior, interests, or activities. These symptoms are typically present in the early developmental period and bring significant impairment to individuals’ lives. In addition to these primary symptoms, hyper- or hypo-reactivity to sensory input have been added to the diagnostic criteria in the Diagnostic and Statistical Manual of Mental Disorders (DSM-5) [1–3]. Sensory issues in ASD include difficulties adapting to temperature changes, discomfort with specific sounds, textures, or smells, attachment to moving objects, and aversion to light, and these symptoms often manifest simultaneously [4–9].

Sensory problems associated with ASD were first reported by Kanner [10], who is regarded as the pioneer of research on ASD. Since his report, numerous studies have indicated that 65% to 92% of ASD patients have some form of sensory problems [11–14]. Among these symptoms, hypersensitivity to light stimuli (photophobia) is a characteristic feature of the visual perception of ASD patients, who have made complaints like “switching between lightness and darkness is uncomfortable” and “flashing light is uncomfortable” [15,16]. If there is no lesion in the opaque media, photophobia may stem from an issue with pupillary response, which regulates the appropriate amount of light.

Photophobia is primarily caused by excessive light stimulation. It occurs when pupillary constriction is impaired or the pupil is larger than appropriate for the light intensity. The pupil diameter is determined by the pupillary dilator muscle (causing mydriasis) and the pupillary sphincter muscle (causing miosis), which are respectively controlled by the sympathetic and parasympathetic nerves. These autonomic nerve functions, which have an antagonistic relationship, can be evaluated by measuring pupillary response [17,18]. It is well known that ASD is accompanied by an imbalance in the autonomic nervous system, and pupillometry is often utilized for non-invasive evaluation of the autonomic nervous system. Indeed, studies investigating pupillary light reflexes (PLR) in ASD have consistently shown reduced pupillary constriction compared to typically developing (TD) [19–22]. However, the background of photophobia involves the possibility of both weak miosis and excessive mydriasis, which cannot be explained by observations of PLR in the previous studies.

In a systematic review and meta-analysis, adults with ASD were found to be at increased risk of several co-occurring mental health conditions, including anxiety, depression, and social-phobia disorders. While the reported lifetime prevalence of these conditions varied across studies, they were consistently found to be particularly prominent in adults with ASD [23,24]. Additionally, high rates of sleep-wake disorders, psychosomatic gastrointestinal problems, elimination disorder, and ADHD were observed, highlighting the complexity of their mental and physical health needs [25,26]. At present, there are no therapeutic medications that specifically address the core symptoms of ASD, as well as sensory issues, including photophobia. Therefore, considering the complexity of ASD, medications such as those for anxiety, depression, and sleep disorders are often prescribed to address specific symptoms or comorbid conditions. While their effectiveness varies greatly between individuals, they are carefully prescribed with the aim of improving overall quality of life [27,28]. Central nervous system medications, such as psychostimulants and antipsychotics, influence the autonomic nervous system and are likely to influence pupil responses as well. However, discontinuing medication for the purpose of pupil measurements is challenging. In this study, we regarded these effects as part of the characteristics of ASD, focusing on pupil responses that may contribute to photophobia.

As mentioned earlier, many previous studies have focused on PLR (miosis) induced by light stimulation, while the process of pupil dilation (mydriasis), which may directly explain another aspect of photophobia in ASD, has been largely unexplored. We aim to fill this gap. Thus, in this study, we elucidate the neurological basis of photophobia in ASD by examining 1) pupil diameter regardless of stimuli and 2) pupillary responses not only to bright stimuli but also to dark stimuli in participants with ASD and TD.

## Method

### Ethics statement

This study was approved by the Ethical Committee for Human Psychological Research of Teikyo University (Approval No. 497, 507, and 575). Participants were recruited between 17/9/2019 and 30/7/2020. Those who expressed interest were informed about the study’s objectives and methods, and experiments were conducted with those who provided written consent. Measurement results were immediately stored on a secure hard drive accessible only to researchers, and subsequent analyses were conducted.

### Participants

This study involved 17 participants with ASD (10 males and 7 females) and 23 TD participants (12 males and 11 females). Participants with ASD were recruited through the mailing list of the autistic social community to ensure that only those with a prior diagnosis of ASD from a medical institution were included. TD participants were recruited through a Japanese participant recruitment company (Agekke Inc.) and were carefully selected based on the criteria that they had no history of neurological disease or developmental disorders. Both groups were age-matched, and the mean age and standard error (SE) were 38.7 ±  SE 2.3 for the ASD group and 37.9 ±  SE 2.0 for the TD group, showing no significant difference between the two groups (*p* =  0.783). We also found no significant gender ratio differences (*χ*^2^(1) =  0.01, *p* = .923). Similarly, no significant difference was found in intelligence quotient between ASD (107.4 ±  SE 2.7) and TD (106.4 ±  SE 2.2) groups as measured by the Japanese Wechsler Adult Intelligence Scale (WAIS-III/IV) (*p* =  0.780).

To confirm autistic traits, the Autism Diagnostic Observation Schedule, Second Edition (ADOS-2) was administered to all participant in the ASD group. Each individual’s total score, based on revised diagnostic algorithms, fell within the ASD range (Cut-off criteria: ≧  8) [29]. The Autism-Spectrum Quotient (AQ), a self-report questionnaire measuring autistic traits (Cut-off criteria: > 32) [30,31], was administered to both groups. All TD participants scored below the cut-off (18.6 ±  SE 7.0), while all ASD participants scored above the cut-off (36.0 ±  SE 8.0).

To investigate sensory characteristics, both ASD and TD groups completed the Japanese Version of the Adolescent/Adult Sensory Profile (AASP-J) [32], with scores analyzed using two-sided t-tests.

In addition, we collected information on the medications taken by participants with ASD, as some of these medications could potentially include psychotropic drugs that might influence pupillary responses.

### Stimuli and task

**Fig 1:**
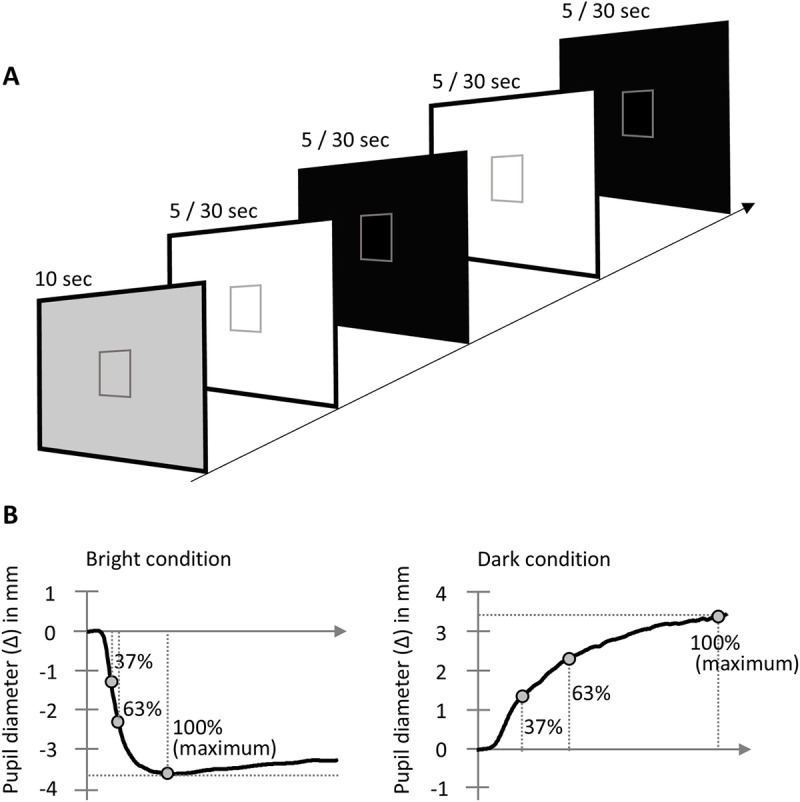
Task design for measurement of pupillary response. (A) The task begins with a dim state, followed by the alternation of bright and dark stimuli every five seconds (Experiment 1) or 30 seconds (Experiment 2). (B) The maximum amplitude (100%) and its latency were identified first, followed by the determination of the amplitudes and corresponding latencies at 37% and 63% of the maximum amplitude.

Stimuli were presented on a 24-inch LCD monitor (22.6° ×  35.1° viewing angle) positioned 60 cm away from the participants using EMR-dStream2 (nac Image Technology). Following a dim state (2.75 cd/m^2^), the bright stimuli (89.03 cd/m^2^) and dark stimuli (0.07 cd/m^2^) were alternately presented. We conducted two experiments with different stimulus durations. In preparation for starting the experiment and to measure the pupil diameter independent of the stimulus, the dim state lasted for ten seconds. Subsequently, bright and dark stimuli were presented for five seconds each (total trials: 24) in Experiment 1, and for 30 seconds each (total trials: 10) in Experiment 2 (Fig 1A). In this study, Experiment 1, which was completed in a shorter in duration (approximately 2 minutes), was conducted first, followed by Experiment 2 (approximately 5 minutes). When the experiment started, a frame (5.1° ×  5.1°) appeared in the center of the monitor to stabilize the gaze during pupil measurements, and participants were required to maintain their gaze within this frame throughout the experiment. Participants’ gaze was constantly monitored by an experimenter in the control room, and if their gaze deviated, the experimenter informed them accordingly.

Prior to both Experiment 1and Experiment 2, a 15-minute period of dark adaptation was carried out. After Experiment 1, we asked for feedback on the experiment. Therefore, Experiment 2 began approximately 20 minutes after the completion of Experiment 1. The dark adaptation and subsequent experiment were consistently conducted in the same soundproof and darkened room, which was separate from the experimenter control room.

### Data recording and analysis

Pupillary responses were measured between 9:00 a.m. and 2:00 p.m., taking into consideration circadian rhythms. For data acquisition, we used an eye tracking system (nac EMR ACTUSⓇ) with a sampling rate of 60 Hz. Data was exported from the device and preprocessing was conducted using adapted R-scripts. We initially applied anti-aliasing and a low-pass filter (5 Hz) to the data, and then segmented it by the dim time, bright trials, and dark trials. Any missing values due to blinks or other factors were replaced with data from the other eye. If the missing values existed for both eyes, a linear interpolation was applied to the data. For the average response of each condition, we excluded the first trial, which showed many eye blinks and eye movements. Diameter during a 100-ms post-stimulus interval served as a baseline correction.

To obtain reliable average pupil responses suitable for evaluation, we adopted data that met the following criteria. 1) To exclude trials with excessive linear interpolation, we rejected trial containing 30% or more missing values within one trial. 2) To ensure the robustness of the average data, we selected data where trials meeting criterion 1 comprised 75% or more of the dataset. 3) Participants who had data meeting criterion 2 in both light and dark conditions were retained.

The data of participants meeting the specified criteria were provided further analysis. We extracted mean pupil diameter corresponding to the dim state (latency, 50–100 ms), which occurred once before the repeated stimuli commenced. Subsequently, we obtained time-series data from the repeated stimuli and generated an average waveform in the bright and dark conditions. In this study, we evaluated not only the maximum amplitude and latency of the pupillary response, but also the response velocity gradient. The amplitude, defined as the amount of change from baseline, was characterized at 100% (maximum), 63%, and 37% levels, with their corresponding times defined as latencies. These parameters were used to analyze changes under the light (pupil constriction) and dark (pupil dilation) conditions, respectively (Fig 1B). Each of these sets of pupil data was compared across the participant groups using two-sided *t*-tests.

## Results

### Sensory profile

**Fig 2:**
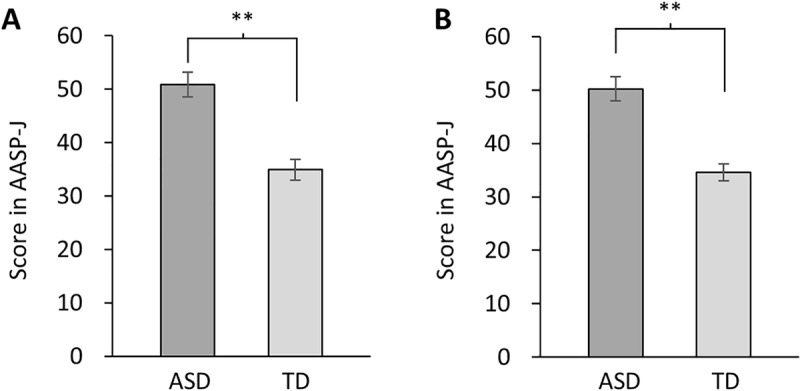
Mean and standard error of sensory properties. (A) ‘Active responding’ and (B) ‘Sensory avoiding’ in AASP-J test. Scores for both parameters were significantly higher in the ASD group compared to the TD group. Significance levels are based on unpaired t-test (**p <  0.01).

The sensory profile in the AASP-J test showed that the “active responding” score was significantly higher in the ASD group (50.9 ±  SE 2.3) than in the TD group (34.9 ±  SE 2.0) (*p* <  0.001) (Fig 2A), and the “sensory avoiding” score was also higher in the ASD group (50.2 ±  SE 2.3) than in the TD group (34.6 ±  SE 1.6) (*p* <  0.001) (Fig 2B). In the AASP-J, ten of the 60 questions are related to visual symptoms, and one specific question is directly related to photophobia. The scores for this question showed a significant difference between the ASD group (3.0 ±  SE 0.5) and TD group (2.0 ±  SE 0.5) (*p* <  0.05). As described later, the number of participants meeting the three-step criteria decreased, particularly in the ASD group. Therefore, we recalculated the scores for participants who met these criteria. In Experiment 1, the ‘active responding’ scores were higher in the ASD group (50.8 ±  SE 2.2) than the TD group (34.0 ±  SE 2.2), and ‘sensory avoiding’ scores were higher in the ASD group (50.5 ±  SE 2.0) than the TD group (33.8 ±  SE 1.6). In Experiment 2, ‘active responding’ scores were higher for the ASD group (49.8 ±  SE 2.3) than the TD group (34.2 ±  SE 2.0), and ‘sensory avoiding’ scores were higher for the ASD group (50.7 ±  SE 1.9) than the TD group (34.1 ±  SE 1.6). In all comparisons, significant differences were observed between the two groups (*p* <  0.001).

### Medications of ASD participants

Of the 17 participants with ASD, 13 (76.4%) were taking some psychotropic agents, of which 11 (84.6%) were taking multiple types of such medication (Table 1). Only three participants were not taking any medication.

**Table 1:** Medication Use Status of ASD Participants (n = 17).

Psychotropic agents		13 (76.4%)	Exp. 1 (n = 8)	Exp. 2 (n = 15)
	Antipsychotics	7 (41.2%)	1	5
	Antidepressants^*^	7 (41.2%)	3	6
	Anxiolytics	4 (23.5%)	1	3
	Hypnotics	7 (41.2%)	4	6
	Anticonvulsants	2 (11.8%)	0	2
	Psychostimulants	5 (29.4%)	2	5
Gastrointestinal agent		5 (29.4%)	2	3

The right columns of the table also include the medications used by ASD participants whose pupillary responses (described later) were utilized.

* All antidepressants were selective serotonin reuptake inhibitors (SSRIs).

### Pupillary responses

**Fig 3:**
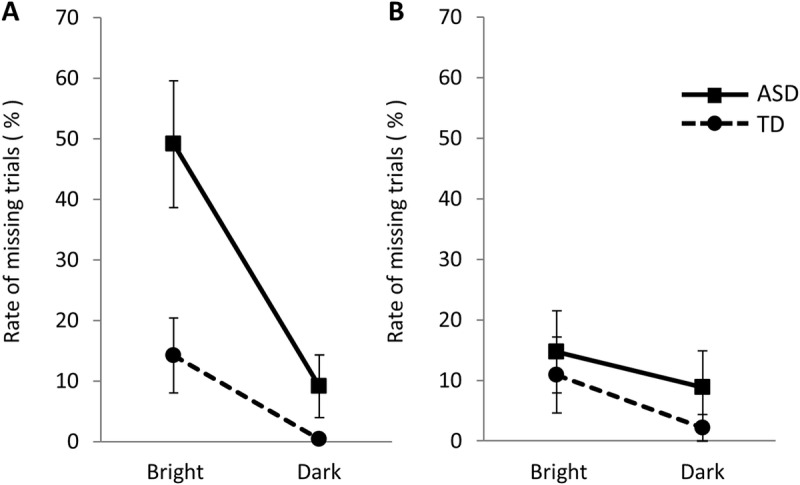
Rate of missing trials. (A) Experiment 1 and (B) Experiment 2. Bars represent standard error. In Experiment 1, the ASD group showed a significantly larger rate of missing trials than the TD group in the bright condition.

In Experiment 1, the participants who met all three-step criteria comprised eight of the 15 in the ASD group (53%; note: Two of the 17 participants did not complete the experiment due to equipment problems) and 20 of the 23 participants in the TD group (87%). In Experiment 2, the number was 15 of 17 in the ASD group (88%) and 20 of the 23 in the TD group (87%). The decrease in the final number of participants is attributed to the high prevalence of missing data under the bright conditions, resulting in a lack of sufficient trials meeting the criteria. In Experiment 1, the rejected trials for the ASD group were 49.1 ±  SE 10.5% in the bright condition and 9.1 ±  SE 5.2% in the dark condition, while these for the TD group were 14.2 ±  SE 6.2% in the bright condition and 0.4 ±  SE 0.4% in the dark condition (Fig 3A). In Experiment 2, the rate of rejected trials in the ASD group was 14.7 ±  SE 6.8% in the bright condition and 8.8 ±  SE 6.0% in the dark condition, while that in the TD group was 10.9 ±  SE 6.3% in the bright condition and 2.2 ±  SE 2.2% in the dark condition (Fig 3B). To examine the effects of group (ASD, TD) and stimulus condition (Bright, Dark), Two-way ANOVA was carried out for each experiment. In Experiment 1, a significant main effect of the group (*F* (1, 72) =  12.84, *p* <  0.001) and stimulus condition (*F* (1, 72) =  16.54, *p* <  0.001) was revealed, while this analysis also identified a group x stimulus condition interaction (*F* (1, 72) =  4.63, *p* <  0.03). The number of rejected trials for the ASD group were significantly larger in the bright condition (*p* <  0.001). On the other hand, no significant main effects were observed in Experiment 2 (group: (*F* (1, 76) =  0.91, *p* = 0.34), stimulus condition: (*F* (1, 76) =  1.91, *p* = 0.17)).

**Fig 4:**
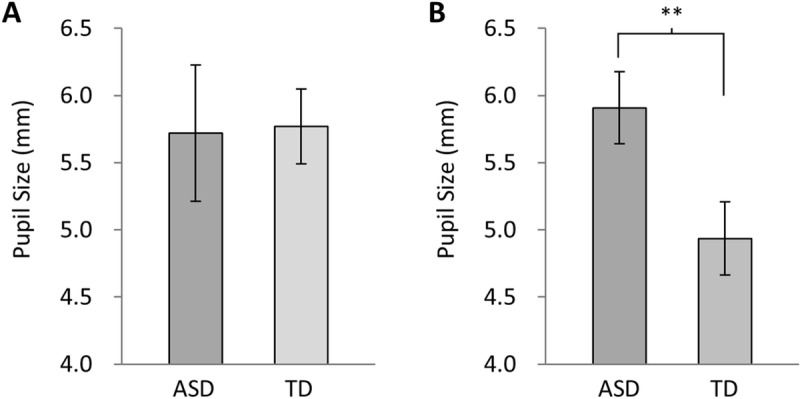
Mean and standard error of pupil diameter in the dim states. (A) Dim state prior to Experiment 1. (B) Dim state prior to Experiment 2. The pupil diameter in the dim state was larger in both the ASD and TD groups during the first experiment (Experiment 1). However, in the second experiment (Experiment 2), the pupil diameter in the TD group showed a significant decrease. Significance levels are based on unpaired t-tests (**p <  0.01).

From the pupillary responses of participants who met the criteria, the pupil diameters in the dim state were measured. The pupil diameter in the dim state prior to Experiment 2 was significantly larger in the ASD group (5.9 ±  SE 0.3 mm) compared to the TD group (4.9 ±  SE 0.3 mm) (*p* =  0.01), while that in Experiment 1 showed no significant difference between the ASD (5.7 ±  SE 0.5 mm) and TD (5.8 ±  SE 0.3 mm) (*p* =  0.8) groups (Fig 4A and 4B). The pupil diameters in Experiment 2, which had 15 participants with ASD, were as follows: Antipsychotics: 5.8 ±  SE 0.6 mm, Antidepressants: 6.3 ±  SE 0.4 mm, Anxiolytics: 6.7 ±  SE 0.5 mm, Hypnotics: 5.6 ±  SE 0.5 mm, Anticonvulsants: 6.4 ±  SE 0.5 mm, and Psychostimulants: 6.3 ±  SE 0.8 mm. Since the participants were taking multiple psychotropic medications, the effects cannot be attributed to a single medication. No significant differences were observed between the medications (*p* >  0.1).

**Fig 5:**
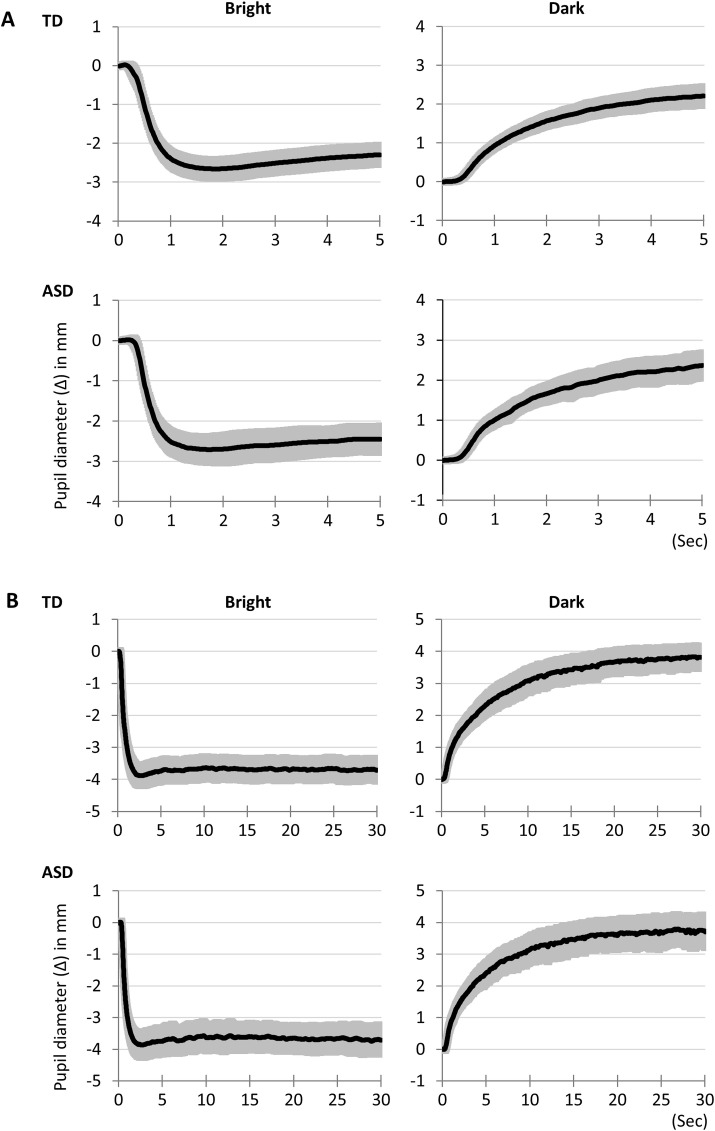
Averaged pupillary responses in bright and dark conditions. (A) Experiment 1 and (B) Experiment 2. Black line indicates average pupillary response. Gray band indicates 95% confidence interval. No obvious differences were observed across the time-series of pupil responses between the ASD and TD groups.

We obtained similar time-series data in both the ASD and TD groups in Experiments 1 and 2, despite not reaching a stable state within the 5-second alternation in Experiment 1 (Fig 5A). Incidentally, no trend of decreasing pupil diameter with repeated bright stimuli was observed in the raw data of participant data. In Experiment 2, where the light/dark stimulus lasted sufficiently (30 seconds) before switching to the dark/light stimulus, a pronounced response was observed (Fig 5B).

Amplitudes of 37%, 63%, and 100%, along with their latencies, were extracted from the time-series data (Table 2). The amplitudes in both bright and dark conditions did not show significant differences between the ASD and TD groups in any of the observed values. These results were consistent in both Experiment 1 and 2. However, significant differences in latency were observed between the ASD and TD groups in the dark condition. The latency to reach 37% amplitude was significantly faster in the ASD group than in the TD group in Experiment 1 (*p* =  0.01), and the latencies to reach 37% and 63% amplitude were significantly faster in the ASD group than in the TD group in Experiment 2 (*p* =  0.03, *p* =  0.03). These results indicate that rapid pupil dilation occurred in ASD under the dark condition.

**Table 2:**
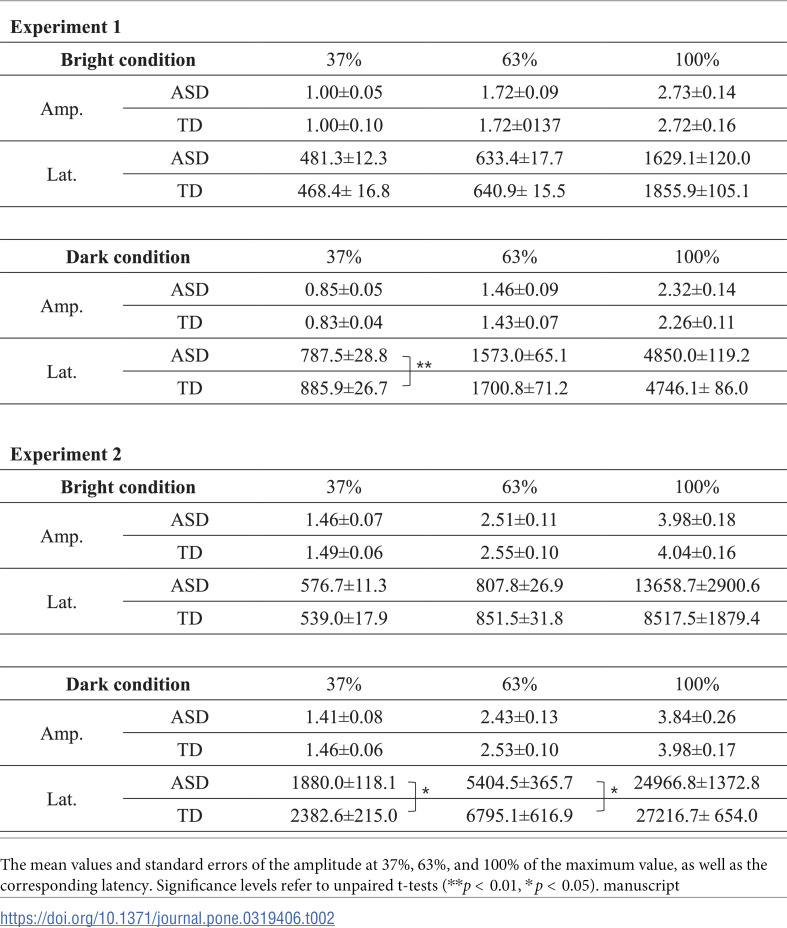
37%, 63%, and 100% amplitude (mm) and latency (ms).

## Discussion

Our findings indicate that pupil diameter in the ASD group tends to be larger regardless of stimuli. Notably, pupil responses in the ASD group showed significant rapid dilation during the dark stimuli compared to the TD group, while there was no significant difference in the maximum amplitude and latency of their pupillary responses to the bright/dark stimuli. This study focused on the neurological background of photophobia in ASD, and the results consistently demonstrated a tendency for pupil dilation in ASD, which may suggest potential new aspects of sympathetic problems to results based on the previous PLR studies [19–22].

Our participants with ASD exhibited sensory abnormalities such as “active responding” and “sensory avoiding” for the AASP-J. ASD individuals tend to dislike brightness and switching between brightness and darkness [15,16]. In our Experiment 1, in which light and dark conditions switched rapidly, the ASD group exhibited a high incidence of missing data, resulting in insufficient trial counts and consequently fewer recruited participants. This suggests that individuals with ASD may struggle in environments where brightness changes rapidly.

When examining pupil diameter in ASD, it is important to consider factors beyond light stimuli, such as viewing distance, arousal levels, attention, and medication effects [17,18,33–37]. In our experiments, a viewing distance of 60 cm involves 1.67 D of accommodation and a pupil diameter adjustment to 5.0–5.5 mm as a result of the parasympathetically driven near reflex [35]. The pupil diameters in the dim state for Experiment 2 of the TD group were within this range. Arousal levels and attention during the experiment were likely controlled through continuous monitoring of participants’ gaze by the experimenter [36]. Regarding the effects of medication, the influence of SSRIs, which have the potential to cause pupil dilation, warrants consideration [37]. The fact that pupil diameter in the dim state among ASD patients remained consistently large suggests that the influence of SSRIs should not be overlooked. However, none of the ASD participants in this study were using SSRIs alone. Therefore, the direct relationship between medications for symptom improvement, pupil dilation, and photophobia should be carefully investigated through future data collection.

Considering these points, our results showing similarly large pupil diameters during the dim state in both the ASD and TD groups in Experiment 1, with the pupil diameter becoming smaller in the TD group in Experiment 2, may indicate sympathetic nervous system arousal in both groups, while this arousal appears to subside in the TD group. It is well-known that mental agitation, anxiety, and stress caused by task load lead to pupil dilation, even in TD individuals [38–40]. Furthermore, in ASD, the sympathetic nervous system has been reported to remain activated even at rest [41], that parasympathetic nervous system weakness and sympathetic nervous hyperexcitability are observed in other physiological indicators such as heart rate variability such as heart rate variability [41–43]. Our results in the dim state suggest that, while still considering the possibility of parasympathetic nervous system vulnerability, ASD exhibited sustained excitation of the sympathetic nervous system.

Our results indicating a rapid pupillary response during the dark stimulus suggest either a radical excitation of the sympathetic nervous system or a weakness of the parasympathetic nervous system. However, the lack of a significant difference in the process of pupillary constriction between the ASD and TD groups in both experiments suggests no major issues with parasympathetic nervous function itself. Previous studies focusing on the PLR in ASD have reported reduced pupil constriction and a sluggish pupil response suggesting delayed parasympathetic activity [19–22]. Our results, seemingly inconsistent with findings from previous studies, prompted us to consider potential contributing factors. The absence of differences in constriction between the ASD and TD groups under bright conditions may be attributed to the intensity of the bright stimuli used in this study (89.03 cd/m²), which may have been substantially lower than in previous studies [19–21]. Although some studies express stimulus intensity as illuminance, making direct comparisons difficult, the lower stimulus intensity in this study may have been less effective for assessing parasympathetic activity, as in previous studies, and more suitable for evaluating sympathetic activity throughout the experiment. Additionally, studies that carefully examined the dilation phase following constriction noted rapid redilation [44], which may share a common mechanism with the rapid dilation observed in our study.

Efficient neuromodulation of glare nerves in the brainstem innervates the pupillary muscles to induce constriction (miosis) and dilation (mydriasis) to adjust to ambient light. The signal of light induces excitation in the Edinger-Westphal nucleus, leading to miosis through the parasympathetic nervous system. Conversely, certain light and darkness trigger excitation in the locus coeruleus, resulting in mydriasis through the sympathetic nervous system. At this time, the locus coeruleus simultaneously sends inhibitory signals to the Edinger-Westphal nucleus, preventing miosis. Thus, the locus coeruleus plays a hub role in switching between mydriasis and miosis [45,46]. Continuous mydriasis in the dim state and rapid mydriasis during the dark conditions in our ASD group both indicate sympathetic overactivity, suggesting potential issues with locus coeruleus function in ASD. Integrating the results of this study with the nervous system of pupillary control suggests that dysregulation with pupillary control by the locus coeruleus could be behind the photophobia in individuals with ASD. As a result, individuals with ASD may not be able to finely adjust pupil diameter to suit the ambient light. Explaining the sensory hypersensitivity in ASD solely through the locus coeruleus-noradrenaline system may be insufficient. However, given that problems with sensory processing often extend beyond vision in ASD, we may have identified a comprehensive neurological background that could explain these issues.

Regarding these results and considerations, we should also consider the effects of blinking. In this study, many blinks were observed, particularly in ASD. While it is natural to close the eyes in response to brightness, opening the eyes against this tendency is also necessary for the experimental task. Opening the eyelids requires contraction of the Müller muscle, controlled by the sympathetic nervous system, and signals from this contraction are fed back to the locus coeruleus. Thus, frequent blinking stimulates the locus coeruleus and activates the sympathetic nervous system [47]. It has been found that eyelid opening itself, rather than visual input, contributes to an increase in arousal level [48]. The large pupil diameter and rapid dilation observed in the ASD group may also be influenced by their frequent blinking and efforts to keep their eyelids open, suggesting the need for further experiments to clarify this point.
